# Molecular eco-epidemiology of *Paracoccidioides brasiliensis* in road-killed mammals reveals *Cerdocyon thous* and *Cuniculus paca* as new hosts harboring this fungal pathogen

**DOI:** 10.1371/journal.pone.0256668

**Published:** 2021-08-24

**Authors:** Bruno de Souza Scramignon-Costa, Fernando Almeida-Silva, Bodo Wanke, Marcelo Weksler, Ricardo Moratelli, Antonio Carlos Francesconi do Valle, Rosely Maria Zancopé-Oliveira, Rodrigo Almeida-Paes, Cecília Bueno, Priscila Marques de Macedo

**Affiliations:** 1 Laboratório de Pesquisa Clínica em Dermatologia Infecciosa, Instituto Nacional de Infectologia Evandro Chagas, Fundação Oswaldo Cruz, Rio de Janeiro, Brazil; 2 Laboratório de Micologia, Instituto Nacional de Infectologia Evandro Chagas, Fundação Oswaldo Cruz, Rio de Janeiro, Brazil; 3 Departamento de Vertebrados, Museu Nacional, Universidade Federal do Rio de Janeiro, Rio de Janeiro, Brazil; 4 Fiocruz Mata Atlântica, Fundação Oswaldo Cruz, Rio de Janeiro, Brazil; 5 Núcleo de Estudos de Vertebrados Silvestres, Universidade Veiga de Almeida, Rio de Janeiro, Brazil; Universidade Federal de Minas Gerais, BRAZIL

## Abstract

Wild animals infected with *Paracoccidioides brasiliensis* represent important indicators of this fungal agent presence in the environment. The detection of this pathogen in road-killed wild animals has shown to be a key strategy for eco-epidemiological surveillance of paracoccidioidomycosis (PCM), helping to map hot spots for human infection. Molecular detection of *P*. *brasiliensis* in wild animals from PCM outbreak areas has not been performed so far. The authors investigated the presence of *P*. *brasiliensis* through nested-PCR in tissue samples obtained from road-killed animals collected nearby a human PCM outbreak spot, Rio de Janeiro state, Brazil and border areas. Eighteen species of mammals were analyzed: *Dasypus novemcinctus* (nine-banded armadillo, n = 6), *Cerdocyon thous* (crab-eating fox, n = 4), *Coendou spinosus* (hairy dwarf porcupine, n = 2), *Lontra longicaudis* (Neotropical river otter, n = 1), *Procyon cancrivorus* (crab-eating raccoon, n = 1), *Galactis cuja* (lesser grison, n = 1), *Tamandua tetradactyla* (collared anteater, n = 1), *Cuniculus paca* (paca, n = 1), and *Bradypus variegatus* (brown-throated three-toed sloth, n = 1). Specific *P*. *brasiliensis* sequences were detected in the liver, spleen, and lymph node samples from 4/6 (66.7%) *D*. *novemcinctus*, reinforcing the importance of these animals on *Paracoccidioides* ecology. Moreover, lymph nodes samples from two *C*. *thous*, as well as lung samples from the *C*. *paca* were also positive. A literature review of *Paracoccidioides* spp. in vertebrates in Brazil indicates *C*. *thous* and *C*. *paca* as new hosts for the fungal pathogen *P*. *brasiliensis*.

## Introduction

Paracoccidioidomycosis (PCM) is a systemic fungal infection occurring in Latin America caused by dimorphic fungi of the genus *Paracoccidioides* whose major known hosts are humans and armadillos. The infection occurs through the inhalation of fungal propagules dispersed in the air after activities involving soil disturbance. The human disease can manifest acutely, which is rare but usually more severe, and chronically, after a long latency period, years or even decades. Its epidemiology is changing over the last three decades, mainly related to changes of interaction between humans and the environment, e.g. migration, deforestation, expansion of agricultural frontiers, and climate changes [1].

In 2010, a cluster of acute PCM cases was described in the hyperendemic area of Botucatu, São Paulo state, Brazil, which was associated to climate changes related to a high intensity El Niño Southern Oscillation [2]. According to an extensive review of PCM epidemiology, no outbreaks of this fungal disease were described up to 2017 [1]. Recently, acute PCM outbreaks have been reported in Rio de Janeiro state, Brazil and in Northeast Argentina, associated with the construction of a new road (Raphael de Almeida Magalhães highway, BR-493) and one of the biggest hydroelectric dams of South America, respectively [3,4].

Considering the traditional predominance of PCM chronic forms (around 90% of the cases) and the great mobility observed in Brazilian populations throughout the country, investigation of environmental sources for PCM infection has been challenging. Despite being a worrisome public health problem, the emergence of acute PCM cases seems to be an opportunity to understand better the process of infection and to identify risk areas, thus helping to promote prevention policies. The recent detection of specific DNA sequences of *Paracoccidioides brasiliensis* in shallow soil samples from the roadside of the acute PCM outbreak area in the state of Rio de Janeiro reinforced the association between the highway construction and the occurrence of these severe acute PCM cases [5]. Moreover, this study discusses that *Paracoccidioides* spp. might be highly associated to nine-banded armadillos (*Dasypus novemcinctus*) burrows. These results supported public health local actions in targeted areas, focused on early clinical recognition and laboratorial diagnosis of these rare and severe acute PCM clinical forms intending to prevent complications, sequelae, and eventually deaths.

Wild animals infected with *Paracoccidioides* spp., especially naturally infected *D*. *novemcinctus*, represent important indicators of the presence of these fungal agents in the environment, particularly considering the great difficulties to isolate the fungus from soil samples and identify it [6]. Thus, the detection of these pathogens in road-killed wild animals has shown to be a key strategy for eco-epidemiological studies of PCM, helping to map risk areas for human infection [7]. As PCM outbreaks had never been identified up to 2017 [3], molecular detection of *P*. *brasiliensis* in wild animals belonging to these hot spot areas has not been performed so far.

The original description of the outbreak in the metropolitan area of Rio de Janeiro involved eight patients diagnosed during a one-year period (December 2015–December 2016) [3]. Since then, until December 2020, 20 additional acute PCM cases from this area were diagnosed at the Evandro Chagas National Institute of Infectious Diseases (INI/Fiocruz), a reference center for clinical assistance and research of PCM in this state (unpublished data). Therefore, besides investigating soil samples, it is important to explore other environmental sources such as animals harboring this fungal pathogen, which could contribute to identify additional high-risk areas for PCM infection.

This study aims to investigate the occurrence of *P*. *brasiliensis* in road-killed wild mammals nearby a PCM outbreak spot in Rio de Janeiro state, Brazil, and border areas. In addition, we intend to contribute to the knowledge of *Paracoccidioides* spp. eco-epidemiology, identifying distribution areas of animal sources from which *P*. *brasiliensis*. have been identified in this study. A literature review of *Paracoccidioides* spp. identification from these animals in the Brazilian territory is also provided.

## Materials and methods

### Study area and animals

The road-killed animals were collected from January to December 2020 in the roadsides of the BR-040, RJ-116, and RJ-122, three routinely monitored highways. The first road connects Rio de Janeiro and Minas Gerais states, both traditional endemic areas for PCM in Brazil. All these three highways cross some municipalities of Rio de Janeiro state where the outbreak of acute PCM has occurred. The Núcleo de Estudos de Vertebrados Silvestres performed the collection and transport of the dead animals, under authorization of the Brazilian Institute of the Environment and Renewable Natural Resources (IBAMA). These animals were placed into plastic bags identified by a code, with date, hour, and geographic position of collection, and sent to the Universidade Veiga de Almeida, where they were maintained in freezers (-20°C) until necropsies were performed, usually a month after collection. For the purposes of this study, we only included not entirely disfigured and recently killed animals (1–10 hours). These animals were defrosted 24 hours before necropsies and their organs (lungs, liver, spleen and mesenteric lymph nodes) were collected and processed for DNA extraction. Maturity stage was based on dental aging, size and weight of the animals [8–15].

#### Ethical statements

The Animal Ethics Committee (Comissão de Ética em Uso de Animais, CEUA/Fiocruz) granted a formal waiver of ethical approval. The Brazilian Institute of the Environment and Renewable Natural Resources (IBAMA) authorized the collection and transport of the biological materials (Abio number 514/2014). The carcasses of the animals used in this study are in accordance with the Operating License number 1187/2013.

#### Molecular detection and identification

The initial step for DNA extraction was performed by grinding the tissue samples frozen by liquid nitrogen using a mortar and pestle [16]. Further steps were carried out in accordance with the manufacturer instructions using the QIAamp DNA Mini Kit (Qiagen, Germany). The DNA pellet was suspended in 200 μl of ultra-pure water and its quality verified through 1% agarose gel electrophoresis. Molecular analyses followed a nested-polymerase chain reaction (nested-PCR) previously described [17], with minor modifications. Briefly, the PCR was performed aiming the rRNA universal fungal region ITS1-5.8S-ITS2 (Internal Transcribed Spacer) using the primers ITS4 (5’-TCCTCCGCTTATTGATATGC-3’) and ITS5 (5’-GGAAGTAAAAGTCGTAACAAGG-3’) for the first amplification; PbITSE (5’-GAGCTTTGACGTCTGAGACC-3’) and PbITSR (5-AAGGGTGTCGATCGAGAGAG-3’) annealing in the ITS-1 and ITS-2 regions for the second, which generates 634 and 387 base pairs (bp) amplicons, respectively. The reactions consisted of 5 μl of DNA in a total volume of 50 μl with final concentrations of 10 mM Tris-HCl (pH 8.3), 50 mM KCl, 2.5 mM MgCl2, 1.5 U of JumpStart *Taq* polymerase (Sigma-Aldrich, St Louis, MO, USA), 10 μM each outer primer, and 100 μM each deoxynucleoside triphosphate (Invitrogen, Thermo Fisher Scientific, Carlsbad, CA, USA). The first reaction conditions were an initial denaturation step at 95°C for 5 min; 35 cycles at 95°C for 30 s, 60°C for 30 s, and 72°C for 1 min; and a final extension step at 72°C for 7 min. The conditions of the second reaction were similar, except for the annealing temperature of 62°C. The PCR products were submitted to a 1.5% agarose gel electrophoresis. DNA was stained with ethidium bromide (0.5 mg/L), and observed using a UV transiluminator. DNA extraction and nested-PCR experiments were performed in triplicate for each organ of all included animals. DNA bands around 387 bp were excised from the gel, purified with the Illustra^TM^ GFX^TM^ PCR DNA and Gel Band Purification Kit (GE Healthcare, Buckinghamshire, UK). The nucleotide sequences were determined via automatic capillary Sanger sequencing at the sequencing platform of the Fundação Oswaldo Cruz—PDTIS/Fiocruz, using the ABI 3730xl- Applied Biosystems machine and the BigDye Terminator v3.1 cycle sequencing kit (Thermo Fisher Scientific, Waltham, MA, USA). Sequences from both DNA strands were generated and edited with the Sequencher software version 4.6 (Gene Codes Corporation, United States). Contiguous sequences were assembled and had their consensus extracted. Then, they were aligned using the ClustalW algorithm [18] with the MEGA software version 6.0, and compared with sequences deposited in the NCBI database (http://www.ncbi.nlm.nih.gov/BLAST) through BLAST search [19]. The sequences generated in this study were deposited in the same database.

#### Literature review

In order to identify other studies describing the occurrence of *Paracoccidioides* spp. in vertebrates in Brazil, a review of the literature was conducted in three different databases: Pubmed/MEDLINE, Scientific Electronic Library Online (SciELO), and Web of Science. The search strategy included a combination of keywords: *Paracoccidioides* [AND] Brazil [AND] animals, mammals, vertebrates. The variables collected included the animals’ Order, Family, Species, the methodology of fungal identification, and the animals’ place of origin.

#### Cartographic analysis

Thematic maps including the geographic positions of the animals evaluated in this study and the distribution of the same animals’ sources from which *Paracoccidioides* spp. was identified in the literature was created using the QGIS 3.14.15 software. Base maps were obtained from the Brazilian Institute of Geography and Statistics (IBGE).

## Results

Eighteen animals were included in this study. Data obtained from these animals, including their identification, sex, maturity stage, species, cities and GPS coordinates of the collection sites are detailed in Table 1.

**Table 1:** Data of the road-killed animals analyzed in this study including their sex, maturity, species, locality and global positions where they were collected, and nested-PCR results from samples of different organs.

Identification	Sex	Maturity	Species	City, state	GPS		Nested-PCR results			
					Latitude	Longitude	Lung	Liver	Spleen	MLN
**CBPNT 32**	F	Adult	*Bradypus variegatus*	Rio de Janeiro, RJ	Unavailable	Unavailable	˗	˗	˗	˗
**CBRJ 116–59** *	M	Adult	*Cerdocyon thous*	Cachoeiras de Macacu, RJ	22°37’20.8"S	42°43’31.6"W	˗	˗	˗	+
**CB1550** *	M	Adult	*Cerdocyon thous*	Matias Barbosa, MG	21°51’58.0"S	43°21’31.4"W	˗	˗	˗	+
**CB1564**	M	Adult	*Cerdocyon thous*	Três Rios, RJ	22°08’22.5"S	43°09’31.7"W	˗	˗	˗	˗
**CB1581**	M	Adult	*Cerdocyon thous*	Petrópolis, RJ	22°20’04.0"S	43°07’59.0"W	˗	˗	˗	˗
**CB1544**	M	Sub-adult	*Coendou spinosus*	Petrópolis, RJ	22°34’21.0"S	43°15’21.0"W	˗	˗	˗	˗
**CBRJ 116–55**	F	Young	*Coendou spinosus*	Cachoeiras de Macacu, RJ	22°30’55.0"S	42°41’42.1"W	˗	˗	˗	˗
**CB1554** *	F	Adult	*Cuniculus paca*	Matias Barbosa, MG	21°49’45.9"S	43°22’22.4"W	+	˗	˗	˗
**CB1504** *	F	Adult	*Dasypus novemcinctus*	Juiz de Fora, MG	21°46’35.0"S	43°25’52.0"W	NT	˗	+	˗
**CB1506** *	M	Adult	*Dasypus novemcinctus*	Petrópolis, RJ	22°31’15.0"S	43°14’11.0"W	NT	+	˗	˗
**CBRJ 116–58**	M	Adult	*Dasypus novemcinctus*	Cachoeiras de Macacu, RJ	22°37’25.8"S	42°43’25.4"W	˗	˗	˗	˗
**CB1547** *	M	Adult	*Dasypus novemcinctus*	Petrópolis, RJ	22°22’38.0"S	43°07’49.0"W	˗	˗	˗	+
**CB1548**	M	Adult	*Dasypus novemcinctus*	Petrópolis, RJ	22°21’09.0"S	43°07’15.0"W	˗	˗	˗	˗
**CB1584** *	F	Adult	*Dasypus novemcinctus*	Areal, RJ	22°12’56.0"S	43°07’53.0"W	˗	˗	+	˗
**CBRJ 122–38**	M	Adult	*Galactis cuja*	Cachoeiras de Macacu, RJ	22°31’49.4"S	42°44’57.0"W	˗	˗	˗	˗
**CB1537**	M	Adult	*Lontra longicaudis*	Simão Pereira, MG	21°57’15.0"S	43°18’01.0"W	˗	˗	˗	˗
**CB1539**	M	Adult	*Procyon cancrivorus*	Matias Barbosa, MG	21°52’48.0"S	43°20’05.0"W	˗	˗	˗	˗
**CBPNT 30**	F	Young	*Tamandua tetradactyla*	Rio de Janeiro, RJ	Unavailable	Unavailable	˗	˗	˗	˗

* Animals with positive nested-PCR results.

RJ = Rio de Janeiro state; MG = Minas Gerais state; NT = not tested; MLN = mesenteric lymph node.

**Fig 1:**
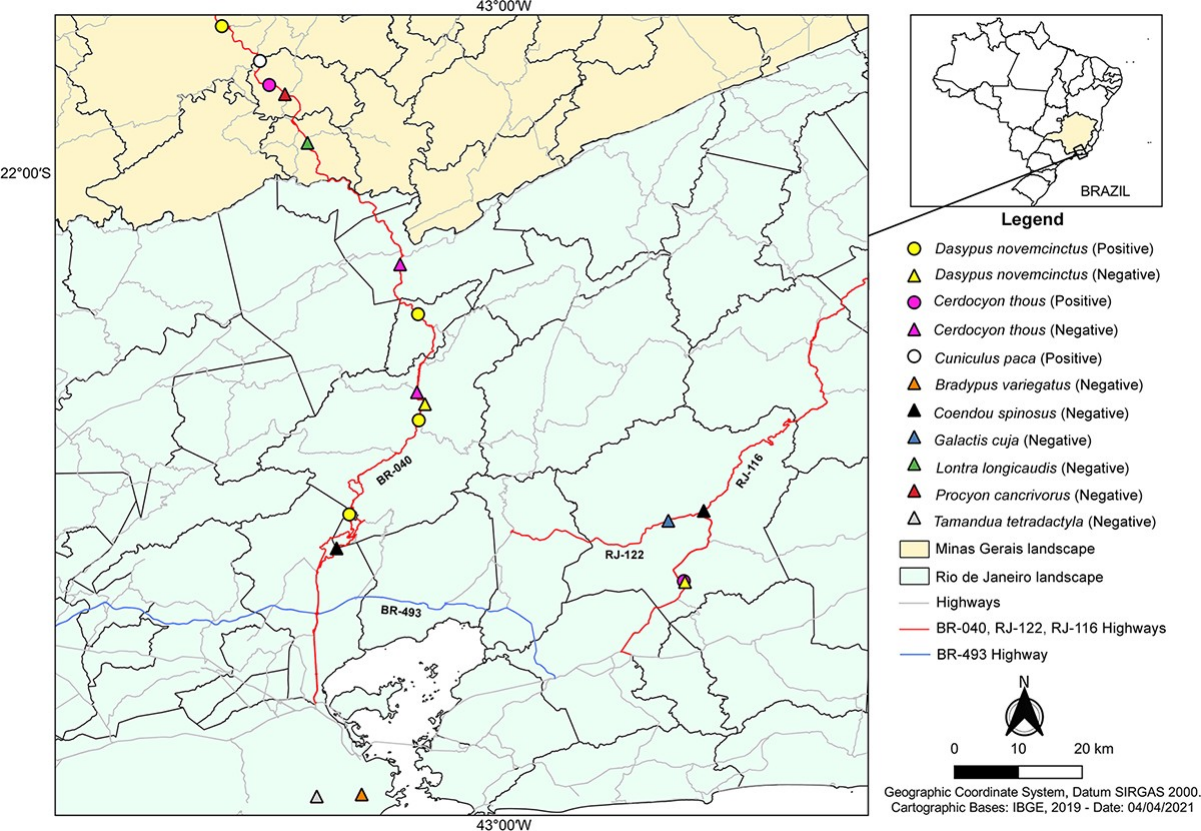
Detection of *Paracoccidioides brasiliensis* in wild animals from this study. Road-killed animals from three highroads in Rio de Janeiro (green) and Minas Gerais (yellow) states were studied. The dark gray lines represent the municipalities’ boundaries, while the light gray lines represent the main highroads of these states. The GPS coordinates of animals’ collection sites are represented by symbols. The color of symbols represents the animal species studied: *Dasypus novemcinctus* (yellow), *Cerdocyon thous* (pink), *Cuniculus paca* (white), *Bradypus variegatus* (orange), *Coendou spinosus* (black), *Galactis cuja* (blue), *Lontra longicaudis* (green), *Procyon cancrivorus* (red), and *Tamandua tetradactyla* (gray). The shape of symbols represents positive (circle) and negative (triangle) nested-PCR results of each animal. Base map retrieved from the Brazilian Institute of Geography and Statistics (IBGE).

Specific *P*. *brasiliensis* sequences were detected in organs from four *D*. *novemcinctus* (CB1504, CB1506, CB1547, CB1584), two *C*. *thous* (CBRJ 116–59, CB1550), and the *C*. *paca* (CB1554) (sequence numbers MZ233470—MZ233476). The sequences generated in this study presented 99.68 to 100% of identity with the ITS sequence of *P*. *brasiliensis* (MN519724.1). The positivity of the organs evaluated from each animal is also depicted in Table 1. A thematic map illustrating the sites of animal collection from this study, together with their nested-PCR results is shown in Fig 1.

**Fig 2:**
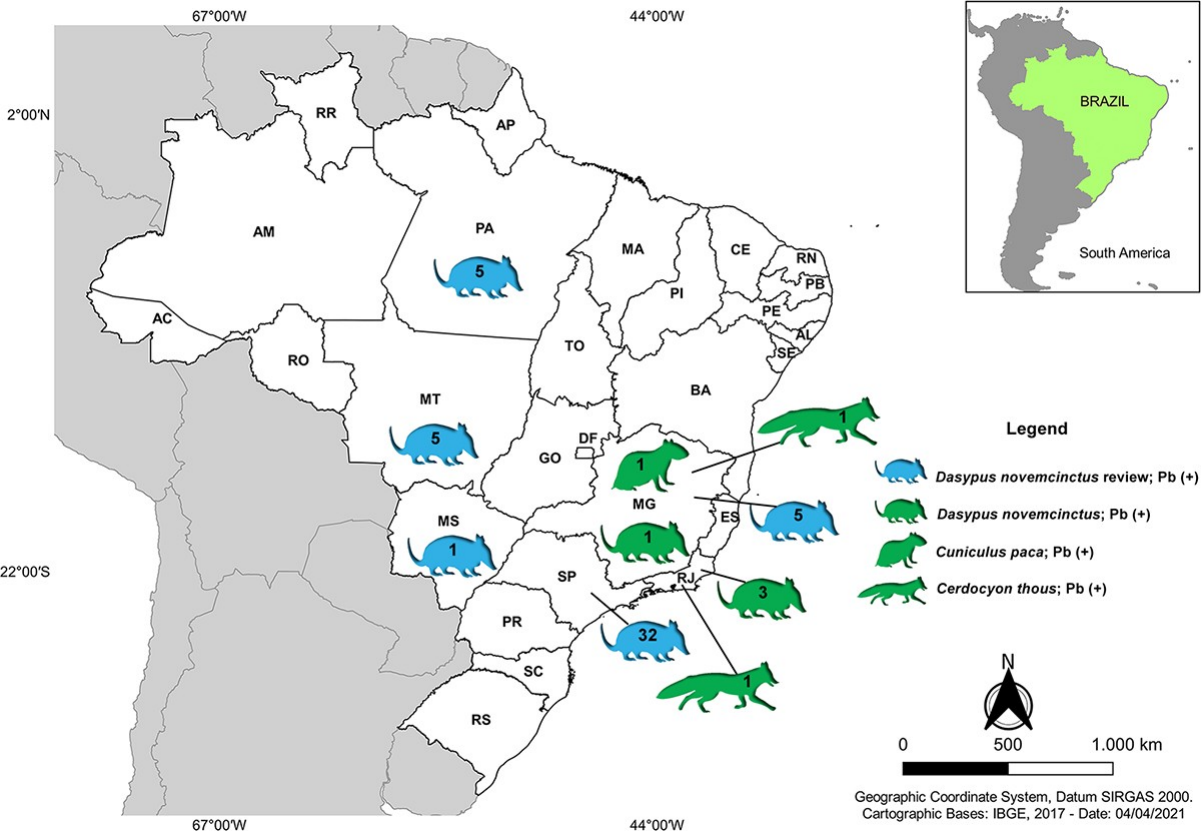
Distribution of *Dasypus novemcinctus*, *Cuniculus paca*, and *Cerdocyon thous*, with *Paracoccidioides* spp. molecular detection or isolation in culture across Brazilian states. Data were retrieved from the literature (blue animals) and this study (green animals). Numbers in animals’ symbols represent the amount of animals with positive *Paracoccidioides* results in each state. Base map retrieved from the Brazilian Institute of Geography and Statistics (IBGE).

The literature search about *Paracoccidioides* spp. in vertebrates in Brazil up to July 2021 revealed 20 papers demonstrating visceral detection of these fungal pathogens and 21 papers reporting serological reactiveness or delayed hypersensitivity against *Paracoccidioides* spp. (S1 Table). Among the manuscripts reporting fungal detection, the order Xenarthra prevailed, followed by the order Carnivora and the order Rodentia. Regarding the order Xenarthra, *D*. *novemcinctus* was the species from which *Paracoccidioides* spp. was mostly detected, counting 48 positive nine-banded armadillos. Among the species from the order Carnivora, two presented *Paracoccidioides* spp. detection: four *Canis lupus familiaris* and two *Procyon cancrivorus*, while all the fourteen *C*. *thous* previously investigated did not have detectable levels of *Paracoccidioides* DNA. However, serological reactiveness against *Paracoccidioides* spp. was reported in two crab-eating foxes (*C*. *thous*). Concerning the order Rodentia, *Paracoccidioides* spp. was detected in three members of the family Cricetidae and two of the family Caviidae. No papers reporting attempts of *Paracoccidioides* spp. isolation or detection neither serological investigation in *C*. *paca* were retrieved using our search strategy. Fig 2 depicts the Brazilian states reporting the detection of *Paracoccidioides* spp. from these three species found positive in this study.

## Discussion

The identification of *P*. *brasiliensis* naturally infected *D*. *novemcinctus* reached a turning point in the ecological study of this fungal pathogen, contributing to a better comprehension of its environmental and geographical distribution [20]. Although *Paracoccidioides* spp. ecological niche has not been precisely identified yet, it has been associated with armadillo and their burrows [21], which is also supported by the high frequency of this fungal infection in these animals [22]. *Paracoccidioides* spp. belongs to the order Onygenales, originated 150 million years ago while armadillos integrate the order Xenarthra, which arose in South America during the Paleocene, between 65 and 80 million years ago. This long coexistence over millions of years, notably the intense contact of armadillos with soil and their digging behavior may explain the intimate ecological relationship between them, as well as the fact that armadillos are naturally infected, eventually presenting disease [23,24]. Among the seven animals presenting specific *P*. *brasiliensis* ITS sequences in the present study, four (57%) were nine-banded armadillos, reinforcing the relevance of these animals in the *Paracoccidioides* spp. ecology.

In addition, PCM infection has also been reported in other wild and domestic animals. For this purpose, many studies were conducted, mostly in areas of high PCM endemicity (South and Southeast Brazil), employing different methodologies such as intradermal and serological surveys, histologic and molecular analyzes, and less frequently isolation in culture [20]. Among wild animals, a higher rate of reactiveness to intradermal tests with paracoccidioidin was observed in terrestrial (83%) than arboreal animals (22%) [25]. This is expected considering that the soil is the potential habitat of *Paracoccidioides* spp. and, consequently, the natural source of these fungal pathogens in higher aerial concentrations. In the present study, we observed *P*. *brasiliensis* DNA detection exclusively in terrestrial wild animals (4 armadillos, 2 crab-eating foxes, and 1 paca). However, among all animals analyzed in this work, only one had arboreal habits (*Bradypus variegatus*), probably because terrestrial mammals are more exposed to the risk of a road accident.

Concerning the animals’ scientific order, our results are in accordance with the literature data, showing a higher rate of positivity for identification of *Paracoccidioides* spp. in Xenarthra members, precisely nine-banded armadillos, from which *P*. *brasiliensis* has been detected in several organs [7,22,26]. On the other hand, there are few cases of *P*. *brasiliensis* identification in Carnivora members other than *C*. *thous* (S1 Table) and no reports of this fungal identification in herbivorous rodent pacas. Armadillos present lower corporal temperatures, ranging from 30–35°C, which may facilitate fungal survival, whereas crab-eating foxes and pacas present higher temperature levels, between 37–39°C [7,27]. Surprisingly, we had positive results in lymph node samples from two crab-eating foxes and lung positivity in one paca. This may reflect hot spot PCM areas, where these animals were possibly more exposed to this fungal pathogen.

*Paracoccidioides brasiliensis* detection in the two species herein described (*C*. *thous* and *C*. *paca*) may be underestimated. Remarkably, the habitat use may drives key ecological opportunities for infection in unrelated hosts, especially those who co-existed for long periods with the pathogen. Canids, including the lineage leading to *C*. *thous*, arrived in South America during the last Great American Biotic Interchange during the late Pliocene [28], while *C*. *paca* is a member of the Caviomorph lineage of rodents that arrived in South America in the Eocene [29]; both species are widely distributed in the Brazilian territory [30]. *Cerdocyon thous* and *C*. *paca* have terrestrial habitats and behaviors that may lead to a greater exposure to environmental sources of *Paracoccidioides* spp. For instance, although both present digging behaviors, being capable of tunneling, they usually spend time in dens dug by other animals, notably armadillo burrows aiming to feed, to rest, and self-protect. Therefore, *Paracoccidioides* spp. infection has perhaps been underrated in these hosts. A previous study demonstrated serological reactiveness against *Paracoccidioides* spp. in two crab-eating foxes (*C*. *thous*), which reveals that this species had already been exposed to this fungal pathogen [31]. Serological tools cannot confirm the presence of the pathogen in the host, just a previous contact. On the other hand, molecular methods can detect the pathogen in biological samples [32]. Up to now, there was no evidence in the literature that this host could harbor this fungus. We propose some few hypotheses to explain this matter. First, as previously mentioned, *Paracoccidioides* spp. infection is indeed less expected in warmer blooded mammals. Second, the well-known relationship between *Paracoccidioides* spp. and armadillos may justify a tendency to preferentially include these animals in PCM eco*-*epidemiology studies. Third, studies investigating *Paracoccidioides* spp. in road-killed wild animals are restraint in terms of number and variety of animals included due to the anatomical conditions required to prevent environmental cross contamination. In this study, the authors initially intended to investigate only nine-banded armadillos. However, our investigation has been expanded to include wild animals that forage on the ground as well as those with burrowing and aquatic habits, considering the ideal occurrence of *Paracoccidioides* spp. in higher soil moisture areas [7].

Concerning the geographical origins of the positive animals from this study, most of them habited traditional Brazilian PCM endemic areas in the states of Rio de Janeiro and Minas Gerais [33,34]. Although the results herein presented do not establish a direct association between geographic areas of positive animals and human patients presenting acute PCM related to the previous reported outbreak, we highlight the high level of endemicity of the studied area. In addition, the studied and the outbreak areas share the same climatic conditions and biomes. It is expected that environmental disruptions in these locations may have a significantly higher potential to provoke PCM infection and eventually outbreaks. The high aerial dispersion of fungal infective propagules can possibly reach distant areas, exposing populations of neighbor human settlements or even neighbor cities to the risk of infection and disease. The closer the individuals live or work from disturbed areas, the higher will be the inhaled fungal burden, enhancing the risk of infection.

Some limitations of this study are worth to be mentioned: the major representativeness of animals from certain municipalities may be related to a higher frequency of monitored highways in these places, as well as an elevated incidence of accidents involving wild life in some points of these roads. In addition, the limited number of animals included in the study is justified by the anatomical conditions required to perform the molecular evaluation in order to avoid cross contamination related to environmental sources. Despite all precautions, it worth to mention that negative results of the nested PCR might be due to the long periods of transportation, rottenness or due to bad frosting conditions, degrading fungal cells and DNA. Moreover, it is noteworthy that although the sequencing of ITS region can identify both recognized species of the genus *Paracoccidioides*: *P*. *brasiliensis* and *P*. *lutzii*, the authors herein investigated only *P*. *brasiliensis* due to its high endemicity in the place of the study, whereas *P*. *lutzii* mostly occurs in the Midwest region and around the Amazon region of Brazil [34]. Lastly, it is possible that papers describing *Paracoccidioides* detection in *D*. *novemcinctus*, *C*. *thous* or *C*. *paca* exist in other databases not included in the literature search herein described, or that similar findings of other groups were not published. Even so, this work contributes to improve the knowledge of *Paracoccidioides* spp. ecology, revealing two new potential animal hosts, and also warning about the threat of anthropogenic actions on the nature without an environmental protection plan.

## Supporting information

S1 Table Literature search of studies describing *Paracoccidioides* spp. in vertebrates in Brazil. (XLSX)
